# An association between the endothelial nitric oxide synthase gene G894T polymorphism and premature coronary artery disease: a meta-analysis

**DOI:** 10.18632/oncotarget.20400

**Published:** 2017-08-23

**Authors:** Boqian Zhu, Xinmin Si, Yaoyao Gong, Gaoliang Yan, Dong Wang, Yong Qiao, Bo Liu, Jiantong Hou, Chengchun Tang

**Affiliations:** ^1^ Department of Cardiology, Zhongda Hospital, Southeast University, Nanjing, China; ^2^ Department of Gastroenterology, The First Affiliated Hospital of Nanjing Medical University, Nanjing, China

**Keywords:** premature coronary artery disease, G894T, Glu298Asp, polymorphism, endothelial nitric oxide synthase

## Abstract

Previous epidemiological studies have suggested that genetic factors are more likely to influence the development of premature coronary artery disease (CAD) than disease in older patients. Several studies have evaluated the association between the G894T polymorphism located in an exon of endothelial nitric oxide synthase (eNOS) and the risk of premature CAD. However, the findings were inconsistent. Thus, we performed a meta-analysis to clarify the association; we conducted both overall and subgroup analyses. Odds ratios and 95% confidence interval were calculated to evaluate the association between the G894T polymorphism and the risk of premature CAD. Overall analysis revealed a significant association. Subgroup analysis in terms of ethnicity revealed a significant association, in all models evaluated, between the G894T polymorphism and susceptibility to premature CAD in mixed population. In contrast, no such association was evident in Caucasians and Asians. On further subgroup analysis based on the premature CAD subtypes, we found that the G894T polymorphism was correlated with premature myocardial infarction (MI) but not with premature CAD without MI. In conclusion, we confirmed that the eNOS G894T polymorphism is a risk factor for premature CAD, particularly in those suffering premature MI.

## INTRODUCTION

Coronary artery disease (CAD) is one of the leading causes of morbidity and mortality worldwide [1]. Genetic factors, diabetes mellitus, hypertension, hyperlipidemia, obesity, and smoking are major risk factors for CAD [2]. Premature CAD, defined as CAD developing in males aged < 55 years and females aged < 65 years, has become more prevalent in recent years, particularly in developing countries [3]. Genetic factors seem to play a prominent role in the development of premature CAD [4]. Earlier epidemiological data suggested that such genetic factors were more likely to affect younger than older subjects [3]. Premature CAD is generally associated with a low atherosclerotic burden in the coronary arteries and a family history of CAD [5–6].

Previous studies found that endothelial dysfunction plays a critical role in atherosclerosis progression [7–8]. Endothelial cells produce nitric oxide (NO) synthesized from L-arginine by endothelial nitric oxide synthase (eNOS). NO promotes vasodilation and inhibits platelet aggregation, leukocyte adhesion to the vascular endothelium, oxidation of low-density lipoproteins, and proliferation of vascular smooth muscle cells [9]. Thus, eNOS exerts an atheroprotective role [10].

NO production levels are influenced by several polymorphisms in the eNOS gene located on chromosome 7q35–36; the gene contains 26 exons with a total length of 21 kb, and the encoded protein is responsible for intracellular NO production [11]. Over the past two decades, several eNOS gene polymorphisms have been associated with CAD or myocardial infarction (MI) [12–14].

A variant of the eNOS gene within exon 7, a G→T transversion at nucleotide position 894 of the cDNA, changes Glu298 (GAG) to Asp (GAT) [15]. Several studies have shown that the G894T (Glu298Asp) polymorphism is associated with premature CAD, but the data are conflicting. Therefore, we conducted a meta-analysis to better understand the correlation between the G894T (Glu298Asp) polymorphism and premature CAD development.

## RESULTS

### Characteristics of the included studies

**Figure 1:**
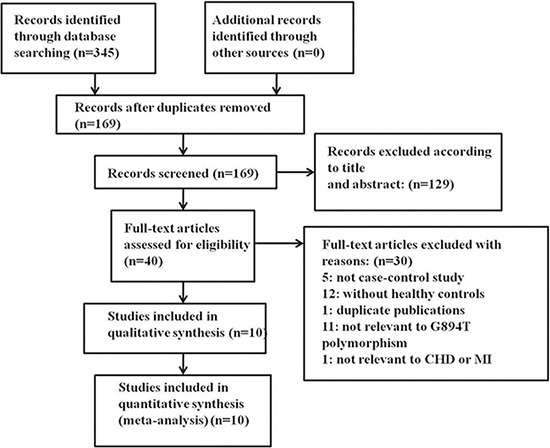
Flow chart of the literature search

A total of 345 relevant studies were identified by the initial search. After full-text review, 335 studies that did not meet the inclusion criteria, contained inadequate data, or were duplicates were excluded. Finally, 10 studies were included [4, 16–24]. The selection process is illustrated in Figure 1. The studies included 1,894 premature CAD patients and 2,096 controls. Of the included studies, five were conducted on Caucasians, two on Asian populations and three on mixed populations. Six studies evaluated premature CAD (premature coronary stenosis) and four premature MI. All genotype distributions were consistent with HWE (*P* > 0.05). The characteristics of the included studies are summarized in Table 1.

**Table 1:** Characteristics of included studies in this meta-analysis

Study	Year	Ethnicity	Case	Genotype						Sample size	% of male (case/control)	Age mean (case/control)	P for HWE	NOS
G894T				Case			Control							
				GG	GT	TT	GG	GT	TT					
Granath	2001	Caucasian	premature CAD	260	248	63	270	287	66	573/624	0.87/0.51	43.9/40.9	0.424	7
Wu	2003	Asian	premature CAD	91	23	0	53	10	2	114/65	0.95/0.83	46.9/49.2	0.108	7
Cam	2005	Caucasian	premature CAD	44	37	34	57	24	2	115/83	0.77/0.78	48.2/44.6	0.777	6
Antoniades	2005	Caucasian	premature MI	97	99	32	255	217	47	228/519	0.93/0.90	46.9/47.2	0.932	8
Vasilakou	2008	Caucasian	premature CAD	109	85	15	76	74	11	209/161	0.83/0.51	52.3/65.6	0.212	6
Isordia-Salas	2010	Mixed	premature MI	104	62	14	134	42	4	180/180	0.75/0.72	39.3/39.7	0.742	8
Jiang	2012	Asian	premature CAD	117	12	2	111	19	1	131/131	0.60/0.60	54.4/53.8	0.851	6
Abdel-Aziz	2013	Mixed	premature CAD	48	46	22	68	39	12	116/119	0.78/0.53	42.4/41.9	0.085	6
Zigra	2013	Caucasian	premature MI	50	46	11	50	42	11	107/103	NA	32.5/31.8	0.626	8
Sampaio	2007	Mixed	premature MI	56	46	13	52	45	7	121/111	0.72/0.67	34.4/33.3	0.509	7

CAD: Coronary artery disease. MI: myocardial infarction. HWE: Hardy-Weinberg equilibrium. NA: not available. NOS: Newcastle-Ottawa scale.

### The overall meta-analysis

**Figure 2:**
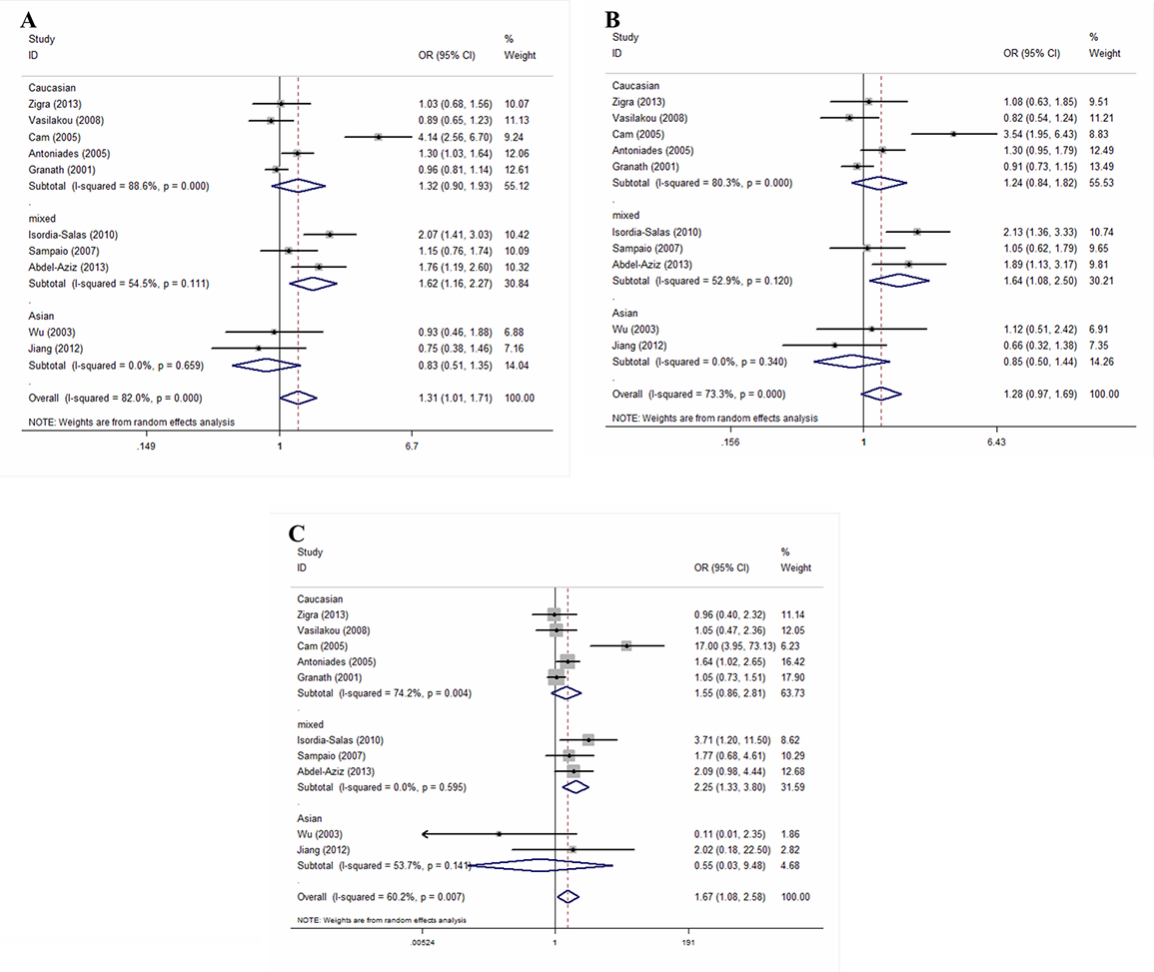
Forest plots of the ORs with 95% CIs, using different genetic models, for the association between the G894T polymorphism and the risk of CAD in patients stratified by ethnicity (**A**) Allelic, (**B**) dominant, and (**C**) recessive models.

Figure 2 presents a summary of the ORs, with corresponding 95% CIs, for the association between the G894T polymorphism and the risk of premature CAD as revealed using the allelic, dominant, and recessive models. We employed a random-effects method when running all models to determine the *P* values for heterogeneity. Overall, we found a significant association between the G894T polymorphism and premature CAD in the allelic (OR = 1.31, 95% CI 1.01–1.71; *P* = 0.045) and recessive (OR = 1.67, 95% CI 1.08–2.58; *P* = 0.022) models, but not in the dominant model (OR = 1.28, 95% CI 0.97–1.69; *P* = 0.09).

### Subgroup analysis

Given that significant heterogeneity was apparent in the overall analysis, we performed subgroup analyses by ethnicity and disease type. Any possible contribution of ethnicity to variations in the overall estimates was evaluated by analyzing the data according to race: Caucasian (five studies recruited 1,238 cases and 1,497 controls), Asian (two studies recruited 245 cases and 196 controls) and mixed population (three studies recruited 411 cases and 403 controls). We found a significant association between the G894T polymorphism and the risk of premature CAD among mixed population when the allelic, dominant, and recessive models were employed. The ORs for the mixed population subgroup were 1.62 (95% CI 1.16–2.27; *P* = 0.005), 1.64 (95% CI 1.08–2.50; *P* = 0.020), and 2.25 (95% CI 1.33–3.80; *P* = 0.003), respectively (Figure 2A–2C). No such association was observed in Caucasians and Asians. The ORs for the Caucasian subgroup were 1.32 (95% CI 0.90–1.93; *P* = 0.160), 1.24 (95% CI 0.84–1.82; *P* = 0.276), and 1.55 (95% CI 0.86–2.81; *P* = 0.145), respectively. The ORs for the Asian subgroup were 0.83 (95% CI 0.51–1.35; *P* = 0.451), 0.85 (95% CI 0.50–1.44; *P* = 0.543), and 0.55 (95% CI 0.03–9.48; *P* = 0.682), respectively (Figure 2A–2C).

**Figure 3:**
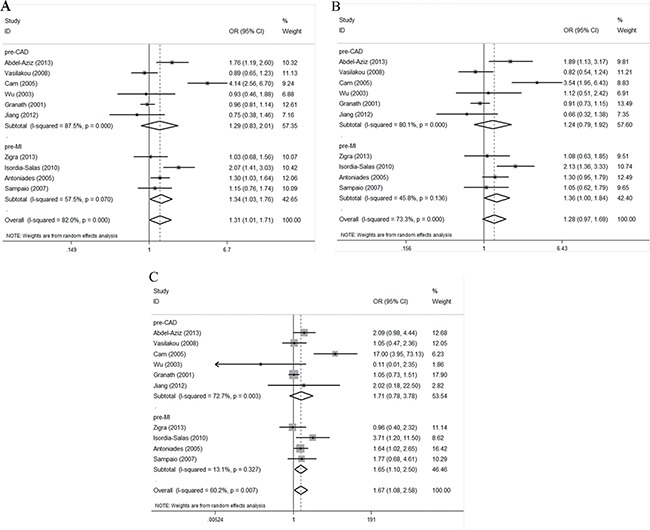
Forest plots of the ORs with 95% CIs, using different genetic models, for the association between the G894T polymorphism and the risk of CAD in patients stratified by type of disease (**A**) Allelic, (**B**) dominant, and (**C**) recessive models.

Upon subgroup analysis by disease type, we found an association between the G894T polymorphism and premature MI susceptibility when all three genetic models were employed. The ORs for the premature MI subgroup were 1.34 (95% CI 1.03–1.76; *P* = 0.032), 1.36 (95% CI 1.00–1.84; *P* = 0.047), and 1.65 (95% CI 1.10–2.50; *P* = 0.017), respectively (Figure 3A–3C). No evidence of any association between the polymorphism and premature CAD susceptibility was apparent. The ORs for the premature CAD subgroup were 1.29 (95% CI 0.83–2.01; *P* = 0.261), 1.24 (95% CI 0.79–1.92; *P* = 0.349), and 1.71 (95% CI 0.78–3.78; *P* = 0.181), respectively (Figure 3A–3C).

### Sensitivity analysis

**Figure 4:**
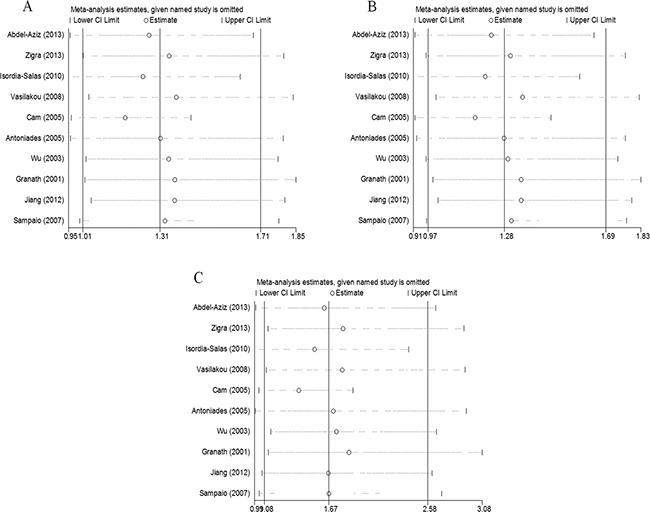
The effect of each study on the overall outcome, as revealed by omitting each study one at a time when various genetic models were evaluated (**A**) Allelic, (**B**) dominant, and (**C**) recessive models.

We assessed the influence of each study on the pooled ORs by omitting the studies one at a time. No study significantly affected the pooled ORs of the allelic, dominant, or recessive models (Figure 4A–4C), confirming the reliability of our results.

### Publication bias

**Figure 5:**
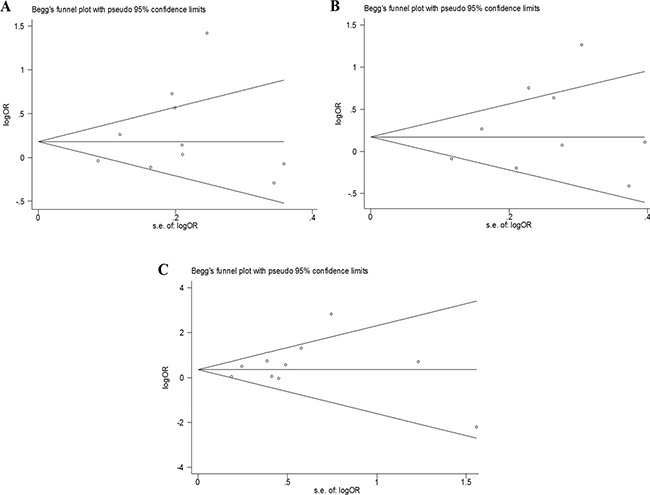
The funnel plots used to assess publication bias (**A**) Allelic, (**B**) dominant, and (**C**) recessive models.

No significant publication bias was detected by Begg's test (allelic model: *P* = 0.721, recessive model: *P* = 0.466, allele analysis: *P* = 0.721) or Egger's regression test (allelic model: *P* = 0.332, recessive model: *P* = 0.326, allele analysis: *P* = 0.362). The funnel plots were not asymmetric (Figure 5A–5C).

## DISCUSSION

CAD is a complex disease influenced by both genetic and environmental factors. Apart from the known traditional risk factors (diabetes mellitus, hypertension, hyperlipidemia, obesity, and smoking), genetic factors also play important roles in pathogenesis and disease development [3, 25]. Those with early-onset CAD constitute a relatively small proportion of all patients with CAD; younger patients account for 5–10% of all MIs [26–27]. However, exploration of the pathogenesis of premature CAD is important; the prognosis is poor and its impact on individuals, families, and societies devastating. Compared with mature CAD, premature CAD is more likely to be influenced by genetic factors [3, 28]. As mentioned above, eNOS plays an atheroprotective role. Several polymorphisms of the eNOS gene have been reported to be associated with CAD risk, among which G894T and T786C are the most clinically relevant [21, 29]. G894T polymorphism results in reduced eNOS enzymatic activity and decreased production of NO by endothelial cells [30]. Reduced eNOS activity causes an increase in smooth muscle cell proliferation after vascular injury, and inhibition of the endothelial NO pathway are present in atherosclerosis [31–32]. Zhang et al. reported eNOS G894T polymorphism may play an important role in CAD development among Asia population [33]. However, reports of the association between the eNOS G894T polymorphism and the risk of premature CAD have been inconsistent.

To the best of our knowledge, our present meta-analysis of 10 studies and 3,990 participants is the first to assess the association between the G894T (Glu298Asp) eNOS polymorphism and the risk of premature CAD. Our overall analysis revealed a significant association between the polymorphism and the risk of premature CAD in all three genetic models evaluated, indicating that the polymorphism may play an important role in the development of premature CAD.

The influence of genetic polymorphisms can be complicated and may differ among various races and geographical regions. Thus, we performed stratification analyses by patient ethnicity. All models revealed a significant association between the G894T polymorphism and premature CAD in mixed populations, but not in Caucasians and Asians. However, only two of the studies focused on Asian populations in this meta-analysis. Further studies are essential to determine whether the observed geographical variability is attributable to differences in genetic backgrounds. Upon further subgroup analyses according to CAD subtype, the G894T polymorphism was correlated with the development of premature MI, but not premature CAD. A possible explanation is that different genetic variants influence the development of coronary atherosclerosis and MI, consistent with the observations of prior studies [34–35]. In addition, the sample sizes of the included studies and the number of studies evaluated may have been too small to allow detection of all potential associations.

Some limitations of our work should be addressed. First, although we collected all eligible studies, the sample sizes were small, particularly in the subgroup analyses, increasing the risk of false-positives or -negatives. Studies with larger sample sizes are required. Second, progression of premature CAD is multifactorial. Given the limited data, we did not analyze our findings in terms of disease, age, or sex. Certain environmental and lifestyle factors may influence the observed associations. The potential roles played by smoking, hypertension, diabetes mellitus, and obesity must be evaluated. Third, although neither the funnel plot nor Egger's test revealed any publication bias, such bias remains possible.

In conclusion, we found that the G894T polymorphism was associated with a risk of premature CAD, particularly in those experiencing premature MI. Thus, testing for the eNOS G894T polymorphism may be useful for clinical assessment and prediction of premature CAD.

## MATERIALS AND METHODS

### Search strategy

Two independent reviewers searched Pubmed, Embase, the Chinese Biomedical Literature Database, and the Cochrane library database (up to December 2016) using the following terms: (‘coronary artery disease’ OR ‘coronary heart disease’ OR ‘myocardial infarction’ OR ‘angina’) AND (‘premature’ OR ‘young’ OR ‘early onset’) AND (‘G894T’ OR ‘Glu298Asp’ OR ‘endothelial nitric oxide synthase’). We perused the reference lists of retrieved articles to identify further potentially relevant studies. Any disagreement was resolved by discussion among the team. We retrieved only texts in English and Chinese. If studies contained overlapping data, only the study with the largest group of subjects, or the most recent study, was included.

### Inclusion and exclusion criteria

The inclusion criteria were studies (1) that assessed the association between the G894T (Glu298Asp) polymorphism and the risk of premature CAD/MI, (2) that were either a case-control or a cohort study, (3) in which all patients had been diagnosed with premature CAD/MI, and the healthy controls did not have CAD, (4) in which the CAD diagnosis was based on coronary angiography and the MI diagnosis on electrocardiography and clinical and laboratory data, and (4) in which the genotype distributions of the controls were in Hardy–Weinberg equilibrium (HWE).

We excluded reviews, case reports, letters, editorials, conference abstracts, and duplicate publications of data from the same study.

### Data extraction

Data were extracted independently by two authors; any disagreement was resolved by discussion among the review team. We extracted the name of the first author, publication year, patient ethnicity, study design, definition of the cases, source of the controls, mean age, age range, male percentage, numbers of cases and controls, genotype distributions of the cases and controls, and the genotyping method used. Authors were contacted with requests for original data when such data were absent from the reports.

### Quality assessment

The quality of eligible studies was assessed by using the Newcastle-Ottawa scale (NOS). NOS quality scores ranged between 0 and 9 stars. Studies with a score ≥ 7 were considered high quality, while studies with a score ≤ 5 were deemed low quality.

### Statistical snalysis

The association between the eNOS G894T polymorphism and the risk of premature CAD was assessed by calculating odds ratios (ORs) with 95% confidence intervals (CIs). We performed an overall comparison as well as a stratification analysis based on patient ethnicity. Each OR and 95% CI were calculated by reference to an allelic model (T vs. G), a dominant model ([GT + TT] vs. GG), and a recessive model (TT vs. [GG + GT]). The chi-squared test was used to determine whether the control genotype distributions were in HWE. Heterogeneity among studies was evaluated by calculation of *Q* test and I^2^ statistics. A *P* value < 0.05 or an *I*^2^ value > 50% indicated that heterogeneity was in play among studies; the random-effects Mantel–Haenszel model was used to compare the data of such studies. Otherwise, the fixed-effects Mantel–Haenszel model was employed. Subgroup analysis was performed by ethnicity and CAD subtype. Publication bias was assessed by drawing a funnel plot and using Egger's test. During the sensitivity analysis, we omitted individual studies to evaluate the reliability of our results. All data were analyzed using STATA 12.0 software for Windows. A *P* value < 0.05 was considered to reflect statistical significance.

## Abbreviations

CAD: coronary artery disease
eNOS: endothelial nitric oxide synthase
NO: nitric oxide
MI: myocardial infarction
HWE: Hardy–Weinberg equilibrium
